# SARS-CoV-2 infection increases risk of acute kidney injury in a bimodal age distribution

**DOI:** 10.1186/s12882-022-02681-2

**Published:** 2022-02-11

**Authors:** Erica C. Bjornstad, Gary Cutter, Pramod Guru, Shina Menon, Isabella Aldana, Scott House, Nancy M. Tofil, Catherine A. St. Hill, Yasir Tarabichi, Valerie M. Banner-Goodspeed, Amy B. Christie, Surapaneni Krishna Mohan, Devang Sanghavi, Jarrod M. Mosier, Girish Vadgaonkar, Allan J. Walkey, Rahul Kashyap, Vishakha K. Kumar, Vikas Bansal, Karen Boman, Mayank Sharma, Marija Bogojevic, Neha Deo, Lynn Retford, Ognjen Gajic, Katja M. Gist, SCCM Discovery VIRUS Investigators Group, Jean-Baptiste Mesland, Pierre Henin, Hélène Petre, Isabelle Buelens, Anne-Catherine Gerard, Philippe Clevenbergh, Rolando Claure-Del Granado, Jose A. Mercado, Esdenka Vega-Terrazas, Maria F. Iturricha-Caceres, Dragana Markotić, Ivana Bošnjak, Oscar Y. Gavidia, Felipe Pachon, Yeimy A. Sanchez, Danijel Knežević, Tanja Kovacevic, Josko Markic, Tatjana Catipovic Ardalic, Branka Polic, Ivo Ivić, Dominko Carev, Robert Glavinic, Mohamed El Kassas, Mohamed Badr, Ahmed Tawheed, Ahmed Tawheed, Hend Yahia, Dimitrios Kantas, Vasileios Koulouras, Sierra-Hoffman, Fernando Valerio, Oscar Diaz, Jose Luis Ramos Coello, Guillermo Perez, Ana Karen Vallecillo Lizardo, Gabina María Reyes Guillen, Helin Archaga Soto, Csaba Kopitkó, Ágnes Bencze, István Méhész, Zsófia Gerendai, Phaneendra Doddaga, Neethi Chandra, Girish Vadgaonkar, Rekha Ediga, Shilpa Basety, Shwetha Dammareddy, Phani Sreeharsha Kasumalla, Umamaheswara Raju, Janaki Manduva, Naresh Kolakani, Shreeja Sripathi, Sheetal Chaitanya, Anusha Cherian, Sreejith Parameswaran, Magesh Parthiban, A. Menu Priya, Chetak Basavaraja, Madhav Prabhu, Vishal Jakati, Puneet Rijhwani, Ashish Jain, Aviral Gupta, Ram Mohan Jaiswal, Ambika Tyagi, Nimish Mathur, Mradul Kumar Daga, Munisha Agarwal, Ishan Rohtagi, Sridhar Papani, Mahesh Kamuram, Kamlesh Kumar Agrawal, Vijendra Baghel, Kirti Kumar Patel, Surapaneni Krishna Mohan, Ekambaram Jyothisree, Mukur Petrolwala, Bharat Ladva, Yuki Itagaki, Akira Kodate, Reina Suzuki, Akira Kodate, Yuki Takahashi, Koyo Moriki, Michihito Kyo, Hidenobu Shigemitsu, Yuka Mishima, Nobuyuki Nosaka, Michio Nagashima, Abdulrahman Al-Fares, Rene Rodriguez-Gutierrez, Jose Gerardo Gonzalez-Gonzalez, Alejandro Salcido-Montenegro, Adrian Camacho-Ortiz, Fatimah Hassan-Hanga, Hadiza Galadanci, Abubakar Shehu Gezawa, Halima M. S. Kabara, Taiwo Gboluwaga Amole, Halima Kabir, Dalha Gwarzo Haliru, Abdullahi S. Ibrahim, Muhammad Sohaib Asghar, Mashaal Syed, Syed Anosh Ali Naqvi, Sidra Ishaque, Ali Faisal Saleem, Naveed Ur Rehman Siddiqui, Salima Sherali, Yasmin Hashwani, Shafia Ishaque, Igor Borisovich Zabolotskikh, Konstantin Dmitrievich Zybin, Sergey Vasilevich Sinkov, Tatiana Sergeevna Musaeva, Marwa Ridha Amer, Mohammed Abdullah Bawazeer, Talal I. Dahhan, Eiad Kseibi, Abid Shahzad Butt, Syed Moazzum Khurshid, Muath Rabee, Mohammed Abujazar, Razan Alghunaim, Maal Abualkhair, Abeer Turki AlFirm, Eiad Kseibi, Syed Moazzum Khurshid, Muath Rabee, Mohammed Abujazar, Razan Alghunaim, Razan K. Alamoudi, Hassan M. AlSharif, Sarah A. Almazwaghi, Mohammed S. Elsakran, Mohamed A. Aid, Mouaz A. Darwich, Omnia M. Hagag, Salah A. Ali, Alona Rocacorba, Kathrine Supeña, Efren Ray Juane, Jenalyn Medina, Jowany Baduria, Mohammed A. Almazyad, Mohammed I. Alarifi, Jara M. Macarambon, Ahmad Abdullah Bukhari, Hussain A. Albahrani, Kazi N. Asfina, Kaltham M. Aldossary, Ana Andrijevic, Srdjan Gavrilovic, Vladimir Carapic, Pedja Kovacevic, Predrag D. Stevanovic, Dejan S. Stojakov, Duska K. Ignjatovic, Suzana C. Bojic, Marina M. Bobos, Irina B. Nenadic, Milica S. Zaric, Marko D. Djuric, Vladimir R. Djukic, Santiago Y. Teruel, Belen C. Martin, Santiago Y. Teruel, Varsha P. Gharpure, Usman Raheemi, Kenneth W. Dodd, Nicholas Goodmanson, Kathleen Hesse, Paige Bird, Chauncey Weinert, Nathan Schoenrade, Abdulrahman Altaher, Esmael Mayar, Matthew Aronson, Tyler Cooper, Monica Logan, Brianna Miner, Gisele Papo, Suzanne Barry, Christopher Woll, Gregory Wu, Erin Carrole, Kathryn Burke, Mustafa Mohammed, Catherine A. St. Hill, Roman R. Melamed, David M. Tierney, Love A. Patel, Vino S. Raj, Barite U. Dawud, Narayana Mazumder, Abbey Sidebottom, Alena M. Guenther, Benjamin D. Krehbiel, Nova J. Schmitz, Stacy L. Jepsen, Lynn Sipsey, Anna Schulte, Whitney Wunderlich, Cecely Hoyt, Abhijit A. Raval, Andrea Franks, Katherine Irby, Ronald C. Sanders, Glenda Hefley, Jennifer M. Jarvis, Anmol Kharbanda, Sunil Jhajhria, Zachary Fyffe, Stephen Capizzi, Bethany Alicie, Martha Green, Lori Crockarell, Amelia Drennan, Kathleen Dubuque, Tonya Fambrough, Nikole Gasaway, Briana Krantz, Peiman Nebi, Jan Orga, Margaret Serfass, Alina Simion, Kimberly Warren, Cassie Wheeler, C. J. Woolman, Amy B. Christie, Dennis W. Ashley, Rajani Adiga, Andrew S. Moyer, George M. Verghese, Andrea Sikora Newsome, Christy C. Forehand, Rebecca Bruning, Timothy W. Jones, Moldovan Sabov, Fatema Zaidi, Fiona Tissavirasingham, Dhatri Malipeddi, Jarrod M. Mosier, Karen Lutrick, Beth Salvagio Campbell, Cathleen Wilson, Patrick Rivers, Jonathan Brinks, Mokenge Ndiva Mongoh, Boris Gilson, Donna Lee Armaignac, Don Parris, Maria Pilar Zuniga, Ilea Vargas, Viviana Boronat, Anneka Hutton, Navneet Kaur, Prashank Neupane, Nohemi Sadule-Rios, Lourdes M. Rojas, Aashish Neupane, Priscilla Rivera, Carlos Valle Carlos, Gregory Vincent, Christopher M. Howard, Cameron McBride, Jocelyn Abraham, Orlando Garner, Katherine Richards, Keegan Collins, Preethi Antony, Sindhu Mathew, Valerie C. Danesh, Gueorgui Dubrocq, Amber L. Davis, Marissa J. Hammers, Ill M. McGahey, Amanda C. Farris, Elisa Priest, Robyn Korsmo, Lorie Fares, Kathy Skiles, Susan M. Shor, Kenya Burns, Corrie A. Dowell, Gabriela “ Hope” Gonzales, Melody Flores, Lindsay Newman, Debora A. Wilk, Jason Ettlinger, Jaccallene Bomar, Himani Darji, Alejandro Arroliga, Alejandro C. Arroliga, Corrie A. Dowell, Gabriela Hope Conzales, Melody Flores, Lindsay Newman, Debora A. Wilk, Jason Ettlinger, Himani Darji, Jaccallene Bomar, Paras B. Khandhar, Elizabeth Kring, Valerie M. Banner-Goodspeed, Somnath Bose, Lauren E. Kelly, Melisa Joseph, Marie McGourty, Krystal Capers, Benjamin Hoenig, Maria C. Karamourtopoulos, Anica C. Law, Elias N. Baedorf Kassis, Allan J. Walkey, Sushrut S. Waikar, Michael A. Garcia, Mia Colona, Zoe Kibbelaar, Michael Leong, Daniel Wallman, Kanupriya Soni, Jennifer Maccarone, Joshua Gilman, Ycar Devis, Joseph Chung, Munizay Paracha, David N. Lumelsky, Madeline DiLorenzo, Najla Abdurrahman, Shelsey Johnson, Maj Andrew M. Hersh, C. P. T. Stephanie L. Wachs, Brittany S. Swigger, C. P. T. Stephanie L. Wachs, Capt Lauren A. Sattler, Capt Michael N. Moulton, Aaron S. Miller, Edwin L. Anderson, Rosemary Nagy, Ravali R. Inja, Pooja A. Nawathe, Isabel Pedraza, Jennifer Tsing, Karen Carr, Anila Chaudhary, Kathleen Guglielmino, Raghavendra Tirupathi, Alymer Tang, Arshad Safi, Cindy Green, Jackie Newell, Katja M. Gist, Imran A. Sayed, John Brinton, Larisa Strom, Kathleen Chiotos, Allison M. Blatz, Giyoung Lee, Ryan H. Burnett, Guy I. Sydney, Danielle M. Traynor, Karissa Nauert, Annika Gonzalez, Mariel Bagley, Anita Santpurkar, Sreekanth Cheruku, Farzin Ahmed, Christopher Deonarine, Ashley Jones, Mohammad-Ali Shaikh, David Preston, Jeanette Chin, Vidula Vachharajani, Abhijit Duggal, Prabalini Rajendram, Omar Mehkri, Siddharth Dugar, Michelle Biehl, Gretchen Sacha, Stuart Houltham, Alexander King, Kiran Ashok, Bryan Poynter, Mary Beukemann, Richard Rice, Susan Gole, Valerie Shaner, Adarsh Conjeevaram, Michelle Ferrari, Narendrakumar Alappan, Steven Minear, Jaime Hernandez-Montfort, Syed Sohaib Nasim, Ravi Sunderkrishnan, Debasis Sahoo, Steven K. Daugherty, Sam Atkinson, Kelly Shrimpton, Sidney Ontai, Brian Contreras, Uzoma Obinwanko, Nneka Amamasi, Amir Sharafi, Sarah Lee, Zahia Esber, Chetna Jinjvadia, Christine Waller, Kara Kallies, Jonean Thorsen, Alec Fitzsimmons, Haley Olsen, Heda R. Dapul, Sourabh Verma, Alan Salas, Ariel Daube, Michelle Korn, Michelle Ramirez, Logi Rajagopalan, Laura Santos, Héctor Collazo Santiago, Ricardo Alan Hernandez, Orma Smalls, Atul Malhotra, Abdurrahman Husain, Qais Zawaydeh, J. H. Steuernagle, Steven Q. Davis, Valentina Jovic, Valentina Jovic, Max Masuda, Amanda Hayes, Kristen Lee Gossett, Jennifer Nason, Sarah Morris, Sarah Deans, Stephanie Houston, Michael Smith, William Snow, Riley Liptak, Hannah Durant, Valerie Pendleton, Alay Nanavati, Risa Mrozowsk, Namrata Nag, Jeff Brauer, Ashwin Dharmadhikari, Sahib Singh, Franco Laghi, Ghania Naeem, Andrew Wang, Kevin Bliden, Amit Rout, Jaime Barnes, Martin Gesheff, Asha Thomas, Melbin Thomas, Alicia R. Liendo, Jovan Milosavljevic, Kenan Abbasi, Nicholas B. Burley, Nicole Rapista, Samuel Amankwah, Sanjay K. Poudel, Saroj Timilsina, Sauradeep Sarkar, Oluwasayo Akinyosoye, Shashi K. Yalamanchili, Sheena Moorthy, Sonia Sugumar, Jonathan Ford, Martin C. Taylor, Charlotte Dunderdale, Alyssa Henshaw, Mary K. Brunk, Jessica Hagy, Shehryar Masood, Sushrutha Sridhar, Manoj K. Gupta, Franscene E. Oulds, Akshay Nandavar, Yuk Ming Liu, Sarah Zavala, Sarah Zavala, Esther Shim, Andy Y. Wen, Allie DaCar, Ronald A. Reilkoff, Julia A. Heneghan, Sarah Eichen, Lexie Goertzen, Scott Rajala, Ghislaine Feussom, Ben Tang, Christine C. Junia, Robert Lichtenberg, Hasrat Sidhu, Diana Espinoza, Shelden Rodrigues, Maria Jose Zabala, Daniela Goyes, Ammu Susheela, Buddhi Hatharaliyadda, Naveen Rameshkumar, Amulya Kasireddy, Genessis Maldonado, Lisseth Beltran, Akshata Chaugule, Hassan Khan, Namrata Patil, Ruhi Patil, Rodrigo Cartin-Ceba, Ayan Sen, Amanda Palacios, Giyth M. Mahdi, Rahul Kashyap, Ognjen Gajic, Vikas Bansal, Aysun Tekin, Amos Lal, John C. O’Horo, Neha N. Deo, Mayank Sharma, Shahraz Qamar, Juan Pablo Domecq, Romil Singh, Alex Niven, Marija Bogojevic, Abigail La Nou, Barbara Mullen, Devang Sanghavi, Pablo Moreno Franco, Pramod Guru, Karthik Gnanapandithan, Hollie Saunders, Zachary Fleissner, Juan Garcia, Alejandra Yu Lee Mateus, Siva Naga Yarrarapu, Nirmaljot Kaur, Abhisekh Giri, Syed Anjum Khan, Juan Pablo Domecq, Nitesh Kumar Jain, Thoyaja Koritala, Alexander Bastidas, Gabriela Orellana, Adriana Briceno Bierwirth, Eliana Milazzo, Juan Guillermo Sierra, Thao Dang, Rahul S. Nanchal, Paul A. Bergl, Jennifer L. Peterson, Travis Yamanaka, Nicholas A. Barreras, Michael Markos, Anita Fareeduddin, Rohan Mehta, Chakradhar Venkata, Miriam Engemann, Annamarie Mantese, Yasir Tarabichi, Adam Perzynski, Christine Wang, Dhatri Kotekal, Adriana C. Briceno Bierwirth, Gabriela M. Orellana, Gerardo Catalasan, Shohana Ahmed, Carlos F. Matute, Ahmad Hamdan, Ivania Salinas, Genesis Del Nogal, Angel Tejada, Anna Eschler, Mary Hejna, Emily Lewandowski, Kristen Kusmierski, Clare Martin, Nasar A. Siddiqi, Lesly Jurado, Lindsey Tincher, Carolyn Brown, Prithvi Sendi, Meghana Nadiger, Balagangadhar Totapally, Bhagat S. Aulakh, Sandeep Tripathi, Jennifer A. Bandy, Lisa M. Kreps, Dawn R. Bollinger, Jennifer A. Bandy, Roger Scott Stienecker, Andre G. Melendez, Tressa A. Brunner, Sue M. Budzon, Jessica L. Heffernan, Janelle M. Souder, Tracy L. Miller, Andrea G. Maisonneuve, Roberta E. Redfern, Jessica Shoemaker, Jennifer Micham, Lynn Kenney, Gabriel Naimy, Holly Balcer, Sara Utley, Dawn Bouknight, Radha Patel, Lama Alfehaid, Majdi Hamarshi, Jeannette Ploetz, Nick Bennett, Kyle Klindworth, Moustafa Younis, Adham Mohamed, Antonia L. Vilella, Sara B. Kutner, Kacie Clark, Danielle Moore, Shina Menon, John K. McGuire, Deana Rich, Howard A. Zaren, Stephanie J. Smith, Grant C. Lewis, Lauren Seames, Cheryl Farlow, Judy Miller, Gloria Broadstreet, Anthony Martinez, Micheal Allison, Aniket Mittal, Rafael Ruiz, Aleta Skaanland, Robert Ross, Umang Patel, Jordesha Hodge, Krunal Kumar Patel, Shivani Dalal, Himanshu Kavani, Sam Joseph, Paul K. Mohabir, Connor G. O’Brien, Komal Dasani, William Marx, Ioana Amzuta, Asad J. Choudhry, Mohammad T. Azam, Neha Gupta, Tracy L. Jones, Shonda C. Ayers, Amy B. Harrell, Brent R. Brown, Utpal S. Bhalala, Joshua Kuehne, Melinda Garcia, Morgan Beebe, Heather Herrera, Chris Fiack, Stephanie Guo, May Vawer, Beth Blackburn, Katherine A. Belden, Michael Baram, Devin M. Weber, Rosalie DePaola, Yuwei Xia, Hudson Carter, Aaron Tolley, Mary Barletta, Mark Steele, Laurie Kemble, Joshua L. Denson, A. Scott Gillet, Margo Brown, Rachael Stevens, Andrew Wetherbie, Kevin Tea, Mathew Moore, Benjamin J. Sines, Thomas J. Bice, Rajany V. Dy, Alfredo Iardino, Jill Sharma, Julia Christopher, Marwan Mashina, Kushal Patel, Erica C. Bjornstad, Nancy M. Tofil, Scott House, Isabella Aldana, Nikhil K. Meena, Jose D. Caceres, Nikhil K. Meena, Sarenthia M. Epps, Harmeen Goraya, Kelsey R. Besett, Ryan James, Lana Y. Abusalem, Akash K. Patel, Lana S. Hasan, Casey W. Stulce, Grace Chong, Ahmeneh Ghavam, Anoop Mayampurath, Dina Gomaa, Michael Goodman, Devin Wakefield, Anthony Spuzzillo, John O. Shinn, Patrick W. McGonagill, Colette Galet, Janice Hubbard, David Wang, Lauren Allan, Aditya Badheka, Madhuradhar Chegondi, Usman Nazir, Garrett Rampon, Jake Riggle, Nathan Dismang, Ozan Akca, Rainer Lenhardt, Rodrigo S. Cavallazzi, Ann Jerde, Alexa Black, Allison Polidori, Haily Griffey, Justin Winkler, Thomas Brenzel, Pauline Park, Andrew Admon, Sinan Hanna, Rishi Chanderraj, Maria Pliakas, Ann Wolski, Jennifer Cirino, Dima Dandachi, Hariharan Regunath, Maraya N. Camazine, Grant. E. Geiger, Abdoulie O. Njai, Baraa M. Saad, Faraaz Ali Shah, Byron Chuan, Sagar L. Rawal, Manal Piracha, Joseph E. Tonna, Nicholas M. Levin, Kayte Suslavich, Rachel Tsolinas, Zachary T. Fica, Chloe R. Skidmore, Renee D. Stapleton, Anne E. Dixon, Olivia Johnson, Sara S. Ardren, Stephanie Burns, Anna Raymond, Erika Gonyaw, Kevin Hodgdon, Chloe Housenger, Benjamin Lin, Karen McQuesten, Heidi Pecott-Grimm, Julie Sweet, Sebastian Ventrone, Murtaza Akhter, Rania Abdul Rahman, Mary Mulrow, Erin M. Wilfong, Kelsi Vela, Markos G. Kashiouris, Tamas Gal, Manasi Mahashabde, Alexandra Vagonis, Rebecca Uber, Haseeb Mahmud, Stefan Leightle, Zoe Zhang, Nicole Vissichelli, Oliver Karam, Alia O’Meara, Heloisa De Carvalho, Katie Rocawich, Ashish K. Khanna, Lynne Harris, Bruce Cusson, Jacob Fowler, David Vaneenenaam, Glen McKinney, Imoh Udoh, Kathleen Johnson, Patrick G. Lyons, Andrew P. Michelson, Sara S. Haluf, Lauren M. Lynch, Nguyet M. Nguyen, Aaron Steinberg, Vishwanath Pattan, Jessica Papke, Ismail Jimada, Nida Mhid, Samuel Chakola

**Affiliations:** 1 Department of Pediatrics, Division of Nephrology, University of Alabama at Birmingham, 1600 7th Avenue South, Lowder Suite 516, Birmingham, AL 35233 USA; 2 Department of Biostatistics, University of Alabama at Birmingham, Birmingham, AL USA; 3 Mayo Clinic, Jacksonville, FL USA; 4 Seattle Children’s Hospital, Seattle, WA USA; 5 Department of Pediatrics, University of Alabama at Birmingham, Birmingham, AL USA; 6 Allina Health (Abbott Northwestern Hospital, United Hospital, Mercy Hospital), Minneapolis, MN USA; 7 MetroHealth Medical Center, Cleveland, OH USA; 8 Beth Israel Deaconess Medical Center, Boston, MA USA; 9 Atrium Health Navicent, GA Macon, USA; 10 Panimalar Medical College Hospital & Research Institute, Chennai, Tamil Nadu India; 11 University of Arizona College of Medicine-Tucson, Tucson, AZ USA; 12 BSES MG Hospital, Mumbai, India; 13 Boston University School of Medicine, Boston, MA USA; 14 Mayo Clinic, Rochester, MN USA; 15 Society of Critical Care Medicine, Mount Prospect, IL USA; 16 University of Colorado Anschutz Medical Campus, Aurora, CO USA

**Keywords:** COVID-19, AKI, Age-spectrum, Hospitalization

## Abstract

**Background:**

Hospitalized patients with SARS-CoV2 develop acute kidney injury (AKI) frequently, yet gaps remain in understanding why adults seem to have higher rates compared to children. Our objectives were to evaluate the epidemiology of SARS-CoV2-related AKI across the age spectrum and determine if known risk factors such as illness severity contribute to its pattern.

**Methods:**

Secondary analysis of ongoing prospective international cohort registry. AKI was defined by KDIGO-creatinine only criteria. Log-linear, logistic and generalized estimating equations assessed odds ratios (OR), risk differences (RD), and 95% confidence intervals (CIs) for AKI and mortality adjusting for sex, pre-existing comorbidities, race/ethnicity, illness severity, and clustering within centers. Sensitivity analyses assessed different baseline creatinine estimators.

**Results:**

Overall, among 6874 hospitalized patients, 39.6% (*n* = 2719) developed AKI. There was a bimodal distribution of AKI by age with peaks in older age (≥60 years) and middle childhood (5–15 years), which persisted despite controlling for illness severity, pre-existing comorbidities, or different baseline creatinine estimators. For example, the adjusted OR of developing AKI among hospitalized patients with SARS-CoV2 was 2.74 (95% CI 1.66–4.56) for 10–15-year-olds compared to 30–35-year-olds and similarly was 2.31 (95% CI 1.71–3.12) for 70–75-year-olds, while adjusted OR dropped to 1.39 (95% CI 0.97–2.00) for 40–45-year-olds compared to 30–35-year-olds.

**Conclusions:**

SARS-CoV2-related AKI is common with a bimodal age distribution that is not fully explained by known risk factors or confounders. As the pandemic turns to disproportionately impacting younger individuals, this deserves further investigation as the presence of AKI and SARS-CoV2 infection increases hospital mortality risk.

**Supplementary Information:**

The online version contains supplementary material available at 10.1186/s12882-022-02681-2.

## Background

The SARS-CoV2 pandemic has killed more than 2.7 million people as of March 2021 [1]. Infection leads to a wide clinical spectrum from asymptomatic to severe multi-organ failure and death. Kidney involvement is increasingly recognized as an important complication of SARS-CoV2 infection, resulting in proteinuria, hematuria, and acute kidney injury (AKI) [2–5]. Kidney involvement is theorized to parallel severity of disease and associated common risk factors of hypoperfusion, ischemia and nephrotoxins. However, another hypothesis for kidney sequelae is related to the virus’ affinity for the ACE2 receptor with high density in the kidney [2, 6].

SARS-CoV2-related AKI has been reported in 25–60% of those critically ill, including up to 37% of critically ill children [7–10]. AKI has been associated with worse outcomes in those with Coronavirus Disease 2019 (COVID-19), the disease caused by SARS-CoV2. Initially, adult hospitals saw a rapid rise in the need for acute dialysis during COVID-19 waves [11, 12], yet this was not seen in pediatric hospitals. Overall, children seem less susceptible to infection and severe disease, so one hypothesis proposes lower rates of AKI/dialysis needs in children is a function of disease severity. Assessment of SARS-CoV2-related AKI across the age spectrum has not previously been reported.

The purpose of this study was to evaluate the incidence and epidemiology of SARS-CoV2-related AKI across the age spectrum and determine if age is an independent risk factor for AKI development in patients hospitalized with SARS-CoV2.

## Methods

### Study Design & Setting

This is a secondary analysis of the observational, international, prospective Viral Infection and Respiratory Illness Universal Study (VIRUS), initiated by Society of Critical Care Medicine (SCCM) in January 2020. VIRUS seeks to ascertain a wide range of clinical and outcome characteristics of patients hospitalized with SARS-CoV2 infection. The unique aspect of this registry is it captures both critically and non-critically ill hospitalized children and adults in the same cohort facilitating comparative evaluations.

Patients included in this analysis were admitted between January 2020 and March 2021; exact admission dates are confidential and not provided to investigators. Detailed methodologies have previously been described [13]. As this was deployed as a rapid registry early in the pandemic, detailed hospital-level characteristics are not available to investigators. Briefly, 298 centers from 26 countries contribute comprehensive pediatric and adult data from hospitalized patients encompassing intensive care units (ICUs) and non-ICUs. Ethical oversight was obtained at each local center and de-identified data stored in REDCap [14].

### Patient population

We evaluated all participants in the registry if they had PCR- or antibody-confirmed presence of SARS-CoV2 infection, complete age and 28-day hospital outcome data, and at least one serum creatinine value. We excluded patients with clinical suspicion but no laboratory confirmation of SARS-CoV2, current pregnancy, chronic dialysis, or chronic kidney disease (CKD) stage 5.

### Potential Bias

As this is an ongoing cohort registry, rapidly deployed during an evolving global pandemic, analyses were conducted by complete case analysis methods which could introduce some biases towards the more severe cases or because of imminent deaths. Nevertheless, the major exclusions were those without creatinine values or missing 28-day hospital outcomes as we assumed these patients to have the least complete data entry and highest risk for potential data entry errors.

### Outcomes

The primary outcome of interest was AKI development as defined by Kidney Disease Improving Global Outcomes (KDIGO) serum creatinine-only criteria within the first 7 days of hospitalization [15]. AKI is defined as a rise in serum creatinine ≥0.3 mg/dL or > 50% from baseline. Urine output is considered part of the KDIGO AKI definition, but the registry data was determined to be insufficient as > 60% of our cohort was missing urine output values. We also further stratified AKI into stages and receipt of dialysis. Additional outcomes of interest included hospital mortality, hospital and ICU length of stay (LOS), and hospital-related complications.

The registry did not capture baseline creatinine (Cr_b_) values (prior to hospitalization). It is therefore standard practice to estimate Cr_b_ [15–17]. However, the estimation of Cr_b_ is not standardized across the age spectrum. Using KDIGO guidelines for adults (≥18 years), we estimated a Cr_b_ by assuming an eGFR of 75 ml/min/1.73m^2^ and back calculating a creatinine with the modification of diet in renal disease (MDRD) equation [15]. No standard international guideline for estimating a Cr_b_ in children exists. We used the validated method of assuming eGFR of 120 ml/min/1.73m^2^ for children 2–17 years and median normative-based eGFR-for-age in children < 2 years and back calculating creatinine with the height-independent equation [18–20]. For patients with CKD, we used the minimum serum creatinine within the first 7 days of hospitalization as Cr_b_ estimation.

Though these are standard assumptions in AKI research in their respective fields of adult and pediatric nephrology [15–17], there is no standard acceptance of estimating Cr_b_ in the transition period from adolescents to adulthood. Therefore, given the lack of standardization for estimating Cr_b_ across the age spectrum, we conducted two sensitivity analyses: [1] using the full age spectrum (FAS) equation for both adults and children that does not assume a fixed eGFR by age but instead changes across the age spectrum to overcome this limitation [21] and [2] using the minimum serum creatinine as an assumed Cr_b_ for all patients. The FAS equation is limited as it has only been validated in Caucasian populations. The assumption of minimum creatinine as a baseline is limited as it assumes all patients return to their baseline within 7 days of hospitalization. In addition, we conducted a sensitivity analysis where race was removed from the MDRD calculation for adults [22].

### Exposure

Primary exposure of interest was age; it was entered as years and months (children< 5 years), years (participants 5–90 years), and limited to ‘> 90’ for those > 90 years of age for privacy. For analysis, those > 90 were classified as 95 years. Age was evaluated as a continuous variable by years and categorical variable by 5-year and 20-year age increments to explore potential non-linear associations.

### Additional variables

As this was an exploratory analysis, we included a variety of additional demographic, pre-hospital, and hospital-related variables from the registry. Sex and race/ethnicity were categorical. The registry de-identified center location except whether the center was in the United States or elsewhere. CDC classifications were used for weight categorization (underweight, normal weight, overweight, obese, severely obese) using BMI data for adults ≥18 years, BMI percentiles for children 2–17 years, and weight-for-height percentiles for children < 2 years [23]. CDC does not provide pediatric classification for severely obese, so those are grouped with obese for those < 18 years. SARS-CoV2 testing was determined by local centers. Other clinical data captured included comorbidities and recent pre-hospital medications as well as inpatient medications within the first 7 days. Comorbidities, including CKD, were determined by medical chart review by local investigators.

Illness severity was categorized by variables that span the age spectrum. Severe illness was defined as a composite of received invasive mechanical ventilation, vasopressor(s) and/or inotrope(s), and/or extracorporeal membrane oxygenation (ECMO). Moderate illness was defined by ICU admission without any organ support therapies listed above. Mild illness was defined as hospitalization but without an ICU admission nor organ support therapies as listed above. As some of these therapies may be clustered within centers, we accounted for this potential in our analyses described below. More traditional markers of illness severity were captured but do not translate across pediatric and adult patients so are not the primary marker assessed in this analysis (e.g., sequential organ failure assessment (SOFA) scores for adults and pediatric risk of mortality (PRISM) scores for children).

### Statistical analyses

Descriptive statistics compared demographic, pre-hospital and inpatient clinical characteristics within the first 7 days of hospitalization among those with and without AKI. Wilcoxon rank sum tests and chi-square tests were used for continuous and categorical variables, respectively. Given the large sample size which leads to highly significant *p*-values, Cohen’s effect size estimates were calculated for continuous variables to better express the magnitude of differences (small effect 0.1–0.3, medium effect 0.3–0.6, large effect > 0.6). Univariate risk differences (RD), odds ratios (OR), and 95% confidence intervals (CIs) were calculated for hospital mortality by AKI stage. To account for common clinical practices, clustering within centers was used via generalized estimating equations (GEE) with logistic regression models to determine if age is an independent risk factor for the development of AKI in SARS-CoV2-related hospitalizations, with adjustments for the potential confounding of sex, hypertension, diabetes mellitus, cancer, CKD, race/ethnicity, and severity of illness as defined above. Determination for potential confounders to include in models were determined by a priori clinical knowledge and directed acyclic graphs. Significance was set at an alpha-level of 0.05. Sensitivity analyses were conducted using different equations for estimating a Cr_b_ and stratifications by comorbidities and whether center was U.S.-based. All analyses were conducted in SAS, version 9.4 (SAS Institute, Inc., Cary, North Carolina).

## Results

### Demographics

**Fig. 1:**
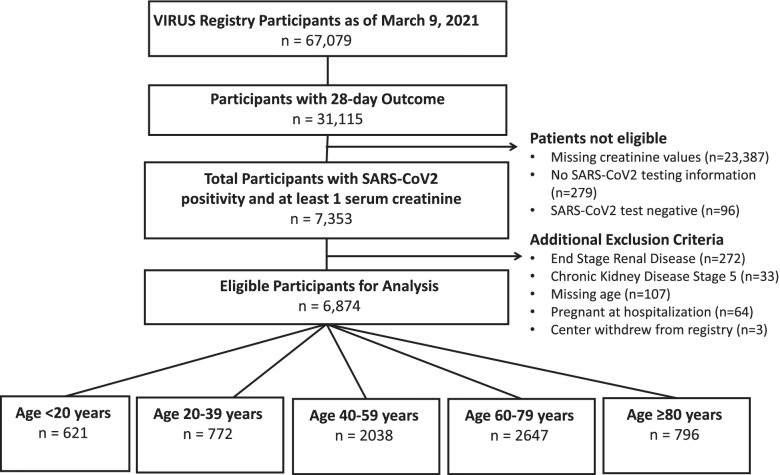
Participant Inclusion Flow Diagram by STROBE Reporting Guidelines

6874 patients from 142 centers met inclusion criteria (Fig. 1). 28% of participants were from non-U.S. centers (Table 1). A total of 39.6% (*n* = 2719) developed AKI within the first 7 days of hospitalization; this was significantly higher among patients in ICUs (1926/4075, 47.3%) compared to non-ICUs (793/2799, 28.3%), *p*-value< 0.0001. Almost 60% of the cohort were admitted to the ICU (*n* = 4075). The median age was 60 years (range 0–95 years) and 9.0% (*n* = 621) were < 20 years (Table 1). Those with AKI were more likely to be older (median age 65 years) than those without AKI (median age 55 years), *p*-value< 0.0001 and effect size 0.45, and more likely to have comorbidities (median 3 versus 1 in those without AKI), *p*-value< 0.0001 and effect size 0.37. Among those < 20 years, 28% (171/621) developed AKI. Differences in AKI risk based on race/ethnicity (*p*-value< 0.0001) were noted. Supplementary Table 1 includes hospital-related associations with AKI.

**Table 1:** Demographics of Participants in VIRUS Registry by AKI status

	Total	No AKI	AKI
	6874	4155 (60.5)	2719 (39.6)
Age, years, median (IQR)	60 (44–71)	55 (39–68)	65 (53–75)
Age Categories			
< 20 years	621 (9)	450 (11)	171 (6)
20 to < 40 years	772 (11)	615 (15)	157 (6)
40 to < 60 years	2038 (30)	1359 (33)	679 (25)
60 to < 80 years	2647 (39)	1375 (33)	1272 (47)
≥ 80 years	796 (12)	356 (9)	440 (16)
BMI category^a^			
Underweight	137 (2)	78 (2)	59 (2)
Normal	1270 (19)	832 (20)	438 (16)
Overweight	1620 (24)	965 (23)	655 (24)
Obesity	1666 (24)	921 (22)	745 (27)
Severe Obesity	552 (8)	302 (7)	250 (9)
*Unknown*	1629 (24)	1057 (25)	572 (21)
Sex (male)^b^	3998 (58)	2327 (56)	1671 (62)
Race/Ethnicity^b^			
White, non-Hispanic	2189 (32)	1273 (31)	916 (34)
White, Hispanic	523 (8)	335 (8)	188 (7)
Black, non-Hispanic	1353 (20)	700 (17)	653 (24)
Black, Hispanic	50 (0.7)	37 (0.9)	13 (0.5)
Asian American	95 (1)	53 (1)	42 (2)
South Asian	1027 (15)	842 (20)	185 (7)
East Asian	36 (0.5)	20 (0.5)	16 (0.6)
West Asian	106 (2)	61 (2)	45 (2)
Other/mixed	845 (12)	511 (12)	334 (12)
White, ethnicity not specified	402 (6)	184 (4)	218 (8)
Black, ethnicity not specified	76 (1)	36 (0.9)	40 (2)
Location of Center			
United States	4984 (73)	2872 (69)	2112 (78)
Non-United States	1890 (28)	1283 (31)	607 (22)
Number of Comorbidities, median (IQR)	2 (1, 4)	2 (1, 4)	3 (1, 5)
Healthy (no comorbidities)	1356 (20)	1020 (25)	336 (12)
Comorbidities^c^			
Hypertension	3404 (50)	1722 (41)	1682 (62)
Diabetes	2279 (33)	1169 (28)	1110 (41)
Heart Disease	1577 (23)	732 (18)	845 (31)
Chronic Kidney Disease	754 (11)	339 (8)	415 (15)
Asthma	757 (11)	478 (12)	279 (10)
Chronic lung disease, not asthma	1395 (20)	770 (19)	625 (23)
Stroke/ Neurological disorder	818 (12)	447 (11)	371 (14)
Cancer	904 (13)	497 (12)	407 (15)
Pre-Hospital Medications			
ACE-I/ARBs	1497 (22)	756 (18)	741 (27)
Diuretics	179 (3)	77 (2)	102 (4)
NSAIDs	610 (9)	377 (9)	233 (9)
Aspirin	1127 (16)	554 (13)	573 (21)
Severity of Disease^d^			
Mild disease	2710 (39)	1950 (47)	760 (28)
Moderate disease	2064 (30)	1389 (33)	675 (25)
Severe disease	2100 (31)	816 (20)	1284 (47)
Ever admitted to ICU (yes)	4075 (59)	2149 (52)	1926 (71)
SARS-CoV2 Testing			
PCR+	6409 (93)	3858 (93)	2551 (94)
Antibody+	98 (1)	45 (1)	53 (2)
PCR and antibody+	367 (5)	252 (6)	115 (4)

Data presented as number (column percentile), except where specified. ACE-I = angiotensin-converting enzyme-inhibitors; *AKI* Acute kidney injury, *ARB* Angiotensin receptor blockers, *BMI* Body mass index, *ECMO* Extracorporeal membrane oxygenation, *ICU* Intensive care unit, *IQR* Interquartile range, *NSAID* Non-steroidal anti-inflammatory drugs, *PCR* Polymerase chain reaction, *VIRUS* Viral Infection and Respiratory Illness Universal Study

^a^BMI Category defined by CDC. Weight-for-height percentiles used for those < 2 years of age, BMI percentiles used for those 2–17 years of age and categorized as underweight for < 5%, normal for 5–85%, overweight for 85–95%, obesity for > 95%. BMI categories for those ≥18 years of age defined as underweight <BMI 18.5, normal BMI 18.5- < 25, overweight BMI 25- < 30, obesity BMI 30- < 40, and severe obesity BMI ≥ 40

^b^Missing data: Sex missing for 1 participant. Race/ethnicity data missing for 172 participants

^c^Multiple comorbidities allowed. Most common ones presented. Heart disease defined as heart failure, coronary artery disease, arrythmias, valvular disease

^d^Severity of disease is defined as: severe disease is a composite of the use of invasive organ support therapy (ventilation, use of vasopressor(s) and/or inotrope(s), and/or use of ECMO); moderate disease is defined as patient admitted to an ICU but did not have any of the invasive organ support therapies as defined for severe disease; and mild disease is defined as neither an ICU admission nor invasive organ support therapies for severe disease

Comparing patients excluded to those included revealed no significant difference by age, sex, or location of center (i.e., U.S.-based). However, those excluded were more likely to have no comorbidities (26%) compared to those included (20%), and only 31% of the excluded group were admitted to the ICU (compared to 59% in this analysis). As expected, those missing creatinine values were often missing other key variables; BMI data missing for 50% of excluded patients compared to 24% of patients in this analysis.

### Hospital complications

Among participants with AKI (*n* = 2719), 64% had Stage 1, 14% Stage 2, 19% Stage 3 without dialysis, and 4% Stage 3 with dialysis (Table 2). Of the patients requiring dialysis, the median duration was 5 days (IQR 2.4–12.4) ranging from 0.2–31.8 days (duration missing for 25/104 patients). Only 7% (*n* = 7) of those who received dialysis in the first week were from non-U.S. centers. Across AKI stages, there was a significant increase in hospital and ICU LOS (effect sizes 0.48 and 0.38, respectively), with the greatest increase being among those receiving dialysis; hospital LOS median 31 days (IQR 22–48) for those on dialysis compared to median 6 days (IQR 4–11) for those with no AKI (*p*-values all < 0.0001). Significant differences across AKI stages were also seen for intubation, new home oxygen requirement on discharge, vasopressor(s)/inotrope(s) use, development of thromboses, and inpatient mortality. The absolute risk of hospital mortality increased significantly (*p*-values< 0.0001) for each AKI stage compared to no AKI. Overall, the OR of hospital mortality in those with AKI compared to those without AKI was 4.0 (95% CI 3.5–4.5). These associations did not change significantly when alternative Cr_b_ estimators were used.

**Table 2:** Hospital Complications by AKI Stages for Patients Admitted with SARS-CoV2 Infection

	Total	No AKI	AKI-1	AKI − 2	AKI-3 (no RRT)	AKI-RRT
	6874	4138 (60.2)	1733 (25.2)	382 (5.6)	517 (7.5)	104 (1.5)
Age Categories						
< 20 years	621 (9)	450 (11)	103 (6)	23 (6)	44 (9)	1 (1)
20 to < 40 years	772 (11)	614 (15)	116 (7)	10 (3)	25 (5)	7 (7)
40 to < 60 years	2038 (30)	1351 (33)	467 (27)	73 (19)	110 (21)	37 (36)
60 to < 80 years	2647 (39)	1369 (33)	773 (45)	191 (50)	259 (50)	55 (53)
≥ 80 years	796 (12)	354 (9)	274 (16)	85 (22)	79 (15)	4 (4)
Admitted to ICU						
Yes	4075 (59)	2136 (52)	1116 (64)	282 (74)	437 (85)	104 (100)
No^a^	2799 (41)	2002 (48)	617 (36)	100 (26)	80 (16)	0 (0)
Hospitalization length of stay (days), median (IQR)^b^	7 (4, 13)	6 (4, 11)	9 (5, 17)	11 (6, 22)	13 (7, 23)	31 (22, 48)
ICU length of stay (days), median (IQR)^b^	5 (2, 11)	4 (2, 9)	6 (2, 13)	8 (2, 18)	6.5 (2.5, 16)	22 (11, 38)
Intubation	1899 (28)	720 (17.4)	596 (34)	185 (48)	298 (58)	100 (96)
Discharged on Oxygen	594 (9)	334 (8)	186 (11)	28 (7)	35 (7)	10 (10)
Vasopressors/ Inotropes	1203 (18)	380 (9)	374 (22)	134 (35)	222 (43)	93 (89)
ECMO	78 (1)	24 (0.6)	32 (2)	10 (3)	10 (2)	2 (2)
Thromboses^c^	337 (5)	140 (3)	112 (7)	22 (6)	40 (8)	23 (22)
Mortality	1314 (19.1)	434 (10.5)	399 (23.0)	157 (41.1)	255 (49.3)	69 (66.4)
RD of Mortality (95% CI)		Reference	12.5% (10.3–14.7)	30.6% (25.6–35.6)	38.8% (34.4–43.2)	55.9% (46.7–65.0)
OR of Mortality (95% CI)		Reference	2.6 (2.2–3.0)	6.0 (4.7–7.5)	8.3 (6.8–10.1)	16.8 (11.1–25.6)

Data presented as number (percentiles), except where specified. *AKI* Acute kidney injury, *AKI-1* AKI stage 1, *AKI-2* AKI stage 2, *AKI-3* AKI stage 3, *CI* Confidence intervals, *ECMO* Extracorporeal membrane oxygenation, *ICU* Intensive care unit, *OR* Odds ratio, *RRT* Renal replacement therapy

^a^Of those never admitted to the ICU, *n* = 139 died (5.0%) and *n* = 30 (1.1%) discharged to hospice care

^b^Length of stay only among survivors (*n* = 5560). Hospital length of stay missing for 91 patients. Intensive care unit length of stay among only those who were ever admitted to ICU and survived (*n* = 2900). ICU length of stay missing for 81 patients

^c^Defined by pre-selected categories of stroke, cerebrovascular accident, deep vein thromboses, and free text entry of the same plus thrombosis, clot, and pulmonary embolism

### Association of age with AKI risk

**Fig. 2:**
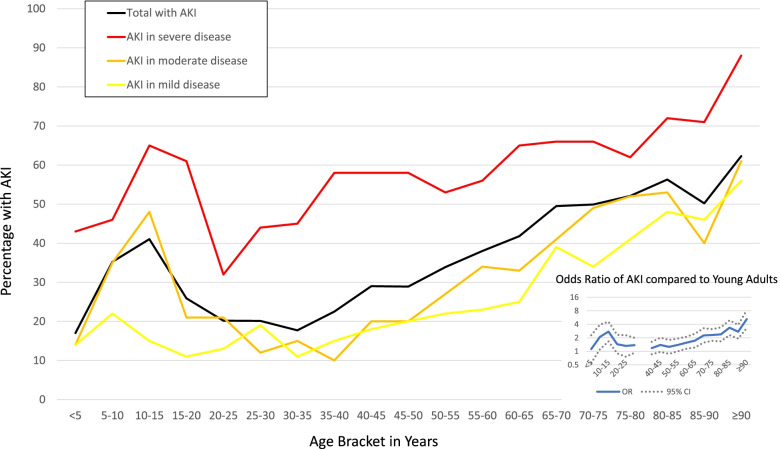
Age Distribution of Hospitalized Patients with SARS-CoV2 who Experienced AKI within First 7 days of Hospitalization. Main figure presents percentage per age bracket who developed acute kidney injury (AKI) among all hospitalized patients and further stratified by severity of illness status. Severe illness is defined as a composite indicator of invasive ventilation, use of vasopressor(s)/inotrope(s), and/or use of extracorporeal membrane oxygenation. Moderate illness is defined as admitted to an intensive care unit but without use of above organ support measures. Mild illness is defined as patient required hospitalization but not in an intensive care unit and without use of above organ support measures. Insert presents the adjusted odds ratio (OR) with 95% confidence intervals (CI) of developing AKI within the first week of hospitalization by age bracket compared to young adults (30–35-year-olds) as the referent category. Adjusted for sex, pre-existing hypertension, diabetes mellitus, cancer, chronic kidney disease, race/ethnicity, and severity of illness. AKI defined per KDIGO guidelines

**Fig. 3:**
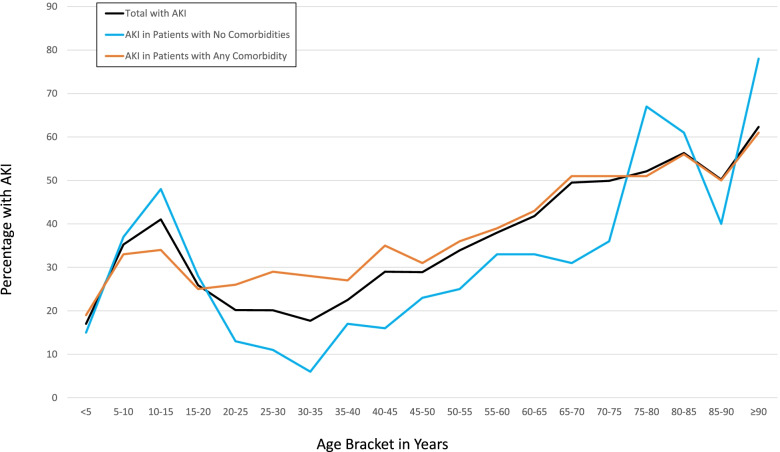
Age Distribution of Hospitalized Patients with SARS-CoV2 who Experienced AKI within First 7 days of Hospitalization Stratified by Presence or Absence of Comorbidities. Presents percentage of hospitalized patients who developed acute kidney injury (AKI) among all hospitalized patients and further stratified by presence of any comorbidity versus no pre-existing comorbidities. AKI defined per KDIGO guidelines

Figure 2 depicts a bimodal distribution of AKI risk by age with those of young adolescence (10–15 years) having a higher risk than both very young children (< 5 years) and older adolescents/young adults (15–35 years), while those over age 65 years also have a high risk of AKI. Even after adjusting for potential confounders (sex, pre-existing hypertension, diabetes mellitus, cancer, CKD, race/ethnicity, and severity of illness) there remains increased risk of AKI in a bimodal distribution (odds ratio inset in Fig. 2). This pattern of AKI distribution did not change when using alternative Cr_b_ estimators (Supplementary Figs. 1, 2, 3 and 4), including the full-age spectrum equation. The pattern of AKI distribution held when evaluating those with no comorbidities versus those with comorbidities (Fig. 3) and again when we evaluated only those in the United States (Supplementary Fig. 5). Table 3 depicts a snapshot of representative age ranges and their adjusted OR of developing AKI in these different scenarios, i.e., by different Cr_b_ estimators and in a population with no pre-existing comorbidities. The data consistently shows an almost 2.5-fold increased odds of developing SARS-CoV2-related AKI for 10–15-year-olds and for 70–75-year-olds when compared to young adults (30–35 years old).

**Table 3:** Adjusted Odds Ratios of Developing AKI by Different Definitions/Populations

Age Bracket	Original	Full-Age Spectrum	Modified MDRD	No Pre-Existing Comorbidities
**10–15-year-olds**	2.74 (1.66–4.56)*	2.49 (1.47–4.22)**	2.66 (1.60–4.41)**	5.35 (2.42–11.81)*
**40–45-year-olds**	1.39 (0.97–2.00)	1.34 (1.00–1.80)***	1.48 (1.03–2.11)***	1.24 (0.65–2.37)
**70–75-year-olds**	2.31 (1.71–3.12)*	2.79 (2.09–3.94)*	2.48 (1.87–3.29)*	2.34 (1.13–4.84)***

Table presents snapshot of odds ratios (95% confidence intervals) for developing acute kidney injury (AKI) compared to 30–35-year-olds. Odds ratios adjusted for sex, race/ethnicity, hypertension, diabetes mellitus, cancer, chronic kidney disease, and severity of illness. Original column defines AKI per KDIGO guidelines when making assumptions about estimating a baseline creatinine. Full-age spectrum column defines AKI per KDIGO guidelines but assumes a more gradual change in eGFR across the age spectrum and uses the previously validated full age spectrum equation to estimate a baseline creatinine. Modified MDRD column defines AKI per KDIGO guidelines when making assumptions about estimating a baseline creatinine, but for adult patients does not include race as a variable in the MDRD equation. The final column only includes hospitalized patients with no pre-existing comorbidities, as such its adjustment model is limited to sex, race/ethnicity, and severity of illness

* *p* < 0.0001

**p < 0.001

***p ≤ 0.05

## Discussion

In a large and diverse cohort evaluating AKI in COVID-19, we found a high incidence of AKI (39.6%) and that it varies across the age spectrum with a bimodal distribution. Given our cohort’s wide age span, we demonstrate a more nuanced view of SARS-CoV2-related AKI than previous evaluations. In every context of our evaluations, there was consistently a bimodal age distribution of AKI risk with the older population and early adolescent (10–15 years) population at higher risk compared to the young adult populations. This is an interesting phenomenon as to date there are only descriptions of a linear relationship between age and COVID-19 severity and its complications [24, 25]. Other known risk factors for AKI were seen in this cohort, such as sex, pre-existing comorbidities (i.e., hypertension, diabetes mellitus, cancer), and race/ethnicity. However, even after controlling for these potential confounders, there remained an association producing a bimodal age distribution in AKI risk; a 10–15-year-old had a similar odds of AKI as a 70–75-year-old (compared to 30–35-year-olds). The bimodal distribution also persisted after controlling for severity of illness and within-center correlations, which suggests something additional is contributing to the AKI risk. This contradicts an early study on SARS-CoV2-related AKI that found illness severity to be the key risk factor for SARS-CoV2-related AKI, but it was a small study (*n* = 223) with results from the early waves (March–June 2020), and excluded children [26]. Interestingly this bimodal distribution differs from previous non-SARS-CoV2 AKI literature which suggests a U-shaped distribution (peaks in infancy and older adults) [27–29].

The differences in AKI risks across the age spectrum found here were not explained by different Cr_b_ estimators. KDIGO is a standard guideline for defining AKI, yet it lacks a standard method for estimating a Cr_b_ in children when one is not known. We therefore evaluated variety of Cr_b_ estimators in pediatric and adult populations. Yet, a bimodal distribution of AKI risk by age remained even with several sensitivity analyses, including a Cr_b_ estimator (FAS) validated across the age spectrum of 2–90 years. The FAS equation assumes a slow transitional change in eGFR from childhood into adulthood [18–20]. In addition, the bimodal age distribution of SARS-CoV2-related AKI development persisted when evaluating only hospitalized patients with no pre-existing comorbidities or evaluating only U.S.-based centers, suggesting that comorbidity differences nor center or country specifics do not explain the bimodal pattern.

The persistence of the bimodal pattern by age, despite multiple iterative analyses, suggests there may be something unique about SARS-CoV2 and its relationship with AKI. One could hypothesize that the propensity of the SARS-CoV2 virus to attack the endothelium could also contribute to the differences seen in the older population and their risk with AKI beyond illness severity [30], though it does not explain the higher risk in early adolescence. There may be a hormonal influence in early adolescence that makes the endothelium more prone to injury compared to younger adults, but this would not fully explain the higher AKI rates in the elderly. We postulate that the bimodal AKI distribution could perhaps be a combination of SARS-CoV2-related vasculopathy and hormonal influences. There may also be yet unknown biological mechanisms that are contributing to this bimodal pattern. For example, we could not account for the different strains or clinical spectrum of SARS-CoV2 presentations which may be an important driver of the bimodal age pattern. A recent report of 2600 hospitalized adults with SARS-CoV2 infection found similarly that high AKI rates are not fully explained by known risk factors and need further exploration [31]. Fully understanding the bimodal age distribution of SARS-CoV2-related AKI risk is even more important now as countries are seeing a shift in age distribution of SARS-CoV2 infections as children are not yet eligible worldwide for vaccinations and new variants may disproportionately affect younger populations. Further in-depth epidemiological studies and animal models may be needed to understand the biological mechanisms underpinning the age distribution in SARS-CoV2-related AKI.

Similar to other studies [7–9, 32], this cohort demonstrates a high rate of AKI in COVID-19 patients; among ICU patients the AKI rate was 47.3% and in non-critically ill patients was 28.3%. Only a few studies report SARS-CoV2-related AKI rates outside of ICUs [33], and our results suggest a high-percentage of non-critically ill patients are at risk.

Other literature has found that SARS-CoV2-related AKI has an increased risk of mortality [4, 8, 9, 34]. In addition to this, we report a strong relationship with mortality and other hospital complications that is proportional to AKI’s severity and seen even in non-critically ill patients and those with mild increases in serum creatinine (≥0.3 mg/dL). Very few reports thus far have explored the complications associated with the varying degrees of AKI severity [2, 10]. This is important as even the slightest degree of AKI may be associated with long-term morbidity and mortality among those hospitalized with SARS-CoV2. Interestingly, though young adolescents had higher risks of AKI compared to middle adulthood, the rates of dialysis were higher in middle adulthood (20–40 years) compared to children (< 20 years). These may be related to center practice differences or the overall small sample of dialysis needs in both of these groups in this cohort (*n* = 7 for 20–40 year-olds and *n* = 1 for < 20 year-olds).

### Limitations

The VIRUS registry has been a real-time assessment of the COVID-19 pandemic, so we may have introduced bias by excluding participants missing data. However, the large sample size provides real-time insight to ongoing trends and allows comparisons across the ages. Comparing the cohort of those with and without creatinine values, we found that we likely had some selection bias toward sicker patients; however, 40% of our participants were never in the ICU. A limitation of evaluating AKI across the age spectrum is the lack of standard Cr_b_ estimators, but our results were similar when using multiple estimators, suggesting there is a true phenomenon of bimodal age distribution in SARS-CoV2-related AKI that deserves further exploration. The registry includes multiple centers and as such risks introducing bias through practice pattern differences between pediatric versus adult centers and regional variations, but we controlled for this in our analyses by accounting for clustering within centers. However, evaluating data from across multiple regions and centers allows a broader view of the epidemiology of SARS-CoV2-related AKI, which is needed to plan for more in-depth case-control or randomized clinical trials evaluating different management and treatment strategies for improved outcomes in SARS-CoV2-related AKI.

## Conclusions

Patients hospitalized with SARS-CoV2 have a high risk of AKI, irrespective of illness severity. We demonstrate an interesting phenomenon of a bimodal age distribution of SARS-CoV2-related AKI risk – high in the elderly and early adolescence – that deserves more in-depth exploration as it was not explained by pre-existing comorbidities, illness severity, eGFR equations, or clustering within centers. Our study reiterates other findings that SARS-CoV2-related AKI at any stage increases patients’ morbidity and mortality. However, as the pandemic lingers, outbreaks will continue, and while younger children remain unvaccinated, it is even more important to understand if there are biological reasons or other unexplored risk factors behind this bimodal age distribution of AKI risk that may guide clinical care improvements in the management of SARS-CoV2 infections and/or provide insights into the pathophysiology of this unique virus.

## Supplementary Information

**Additional file 1: Supplementary Table 1.** Initial Hospital-related Associations with SARS-CoV2 -related AKI. These therapies or complications occur within the first 7 days of hospitalization when SARS-CoV2-related AKI is defined. Data presented as number (column percentiles), except where specified. ACE-I = angiotensin-converting enzyme-inhibitors; AKI = acute kidney injury; ARB = angiotensin receptor blockers; IVIG = intravenous immunoglobulin; NSAID = non-steroidal anti-inflammatory drugs; PRISM = Pediatric Risk of Mortality Score; SOFA = Sequential Organ Failure Assessment. ^a^Initial PRISM score missing for 497 pediatric patients. Baseline SOFA score missing for 2741 adult patients; maximum SOFA score missing for 2016 adult patients.

**Additional file 2: Supplementary Fig. 1.** Age Distribution of Hospitalized Patients with SARS-CoV2 who Experienced AKI within First 7 days of Hospitalization-different baseline creatinine estimators. Main figure presents percentage per age bracket who developed acute kidney injury (AKI) among all hospitalized patients. The original AKI definition (blue) assumes a baseline creatinine based on KDIGO guidelines for adults (eGFR 75 ml/min/1.73m^2^ and back calculates using MDRD equation) and common pediatric definitions assuming an eGFR of 120 ml/min/1.73m^2^ and back calculating using height-independent equation, except for patients with CKD when minimum serum creatinine during first 7 days of hospitalization is assumed to be their baseline creatinine value. Orange line assumes that the minimum creatinine during the first 7 days of hospitalization is the baseline creatinine for all participants. Gray line uses the KDIGO guidelines but back calculates the baseline creatinine for all participants using the FAS equation. Yellow line uses the original definition but uses the MDRD equation minus the race variable. Abbreviations: AKI = acute kidney injury, CKD = chronic kidney disease, eGFR = estimated glomerular filtration rate, FAS = full age spectrum, KDIGO=Kidney Disease Improving Global Outcomes, MDRD = modification of diet in renal disease.

**Additional file 3: Supplementary Fig. 2.** Age Distribution of Hospitalized Patients with SARS-CoV2 who Experienced AKI within First 7 days of Hospitalization-baseline creatinine estimator FAS equation. Main figure presents percentage per age bracket who developed acute kidney injury (AKI) among all hospitalized patients and further stratified by severity of illness status. Severe illness is defined as a composite indicator of invasive ventilation, use of vasopressor(s)/inotrope(s), and/or use of extracorporeal membrane oxygenation. Moderate illness is defined as admitted to an intensive care unit but without use of above organ support measures. Mild illness is defined as patient required hospitalization but not in an intensive care unit and without use of above organ support measures. Insert presents the adjusted odds ratio (OR) with 95% confidence intervals (CI) of developing AKI within the first week by age bracket compared to young adults (30–35-year-olds) as the referent category. Adjusted for sex, race/ethnicity, pre-existing hypertension, diabetes mellitus, cancer, chronic kidney disease, and severity of illness. AKI defined per KDIGO guidelines, but baseline creatinine estimator uses full-age spectrum (FAS) equation for all participants.

**Additional file 4: Supplementary Fig. 3.** Age Distribution of Hospitalized Patients with SARS-CoV2 who Experienced AKI within First 7 days of Hospitalization-baseline creatinine estimator MDRD equation removing race. Main figure presents percentage per age bracket who developed acute kidney injury (AKI) among all hospitalized patients and further stratified by severity of illness status. Severe illness is defined as a composite indicator of invasive ventilation, use of vasopressor(s)/inotrope(s), and/or use of extracorporeal membrane oxygenation. Moderate illness is defined as admitted to an intensive care unit but without use of above organ support measures. Mild illness is defined as patient required hospitalization but not in an intensive care unit and without use of above organ support measures. Insert presents the adjusted odds ratio (OR) with 95% confidence intervals (CI) of developing AKI within the first week by age bracket compared to young adults (30–35-year-olds) as the referent category. Adjusted for sex, race/ethnicity, pre-existing hypertension, diabetes mellitus, cancer, chronic kidney disease, and severity of illness. AKI defined per KDIGO guidelines, but baseline creatinine estimator uses modified MDRD equation removing race component for adults (≥18 years) and height-independent equation for children (< 18 years).

**Additional file 5: Supplementary Fig. 4.** Age Distribution of Hospitalized Patients with SARS-CoV2 who Experienced AKI within First 7 days of Hospitalization-baseline creatinine estimator as minimum serum creatinine. Main figure presents percentage per age bracket who developed acute kidney injury (AKI) among all hospitalized patients and further stratified by severity of illness status. Severe illness is defined as a composite indicator of invasive ventilation, use of vasopressor(s)/inotrope(s), and/or use of extracorporeal membrane oxygenation. Moderate illness is defined as admitted to an intensive care unit but without use of above organ support measures. Mild illness is defined as patient required hospitalization but not in an intensive care unit and without use of above organ support measures. Insert presents the adjusted odds ratio (OR) with 95% confidence intervals (CI) of developing AKI within the first week by age bracket compared to young adults (30–35-year-olds) as the referent category. Adjusted for sex, race/ethnicity, pre-existing hypertension, diabetes mellitus, cancer, chronic kidney disease, and severity of illness. AKI defined per KDIGO guidelines, but baseline creatinine estimator uses minimum serum creatinine value within first 7 days of hospitalization for all participants.

**Additional file 6: Supplementary Fig. 5.** Age Distribution of Hospitalized Patients with SARS-CoV2 who Experienced AKI within First 7 days of Hospitalization Stratified by U.S. versus non-U.S. Hospital. Presents percentage of hospitalized patients who developed acute kidney injury (AKI) among all hospitalized patients and further stratified by hospital center based in the United States versus not in the United States. AKI defined per KDIGO guidelines.

## Data Availability

The datasets used and analysed during the current study are available from the corresponding author/society of critical care medicine on reasonable request.
