# Supplementary material for: SARS-CoV-2 infection increases risk of acute kidney injury in a bimodal age distribution
Source: BMC Nephrol. 2022 Feb 11;23:63. doi: 10.1186/s12882-022-02681-2 (PMC8831033; doi:10.1186/s12882-022-02681-2)
Supplement: Supplementary file 1 — Additional file 1: Supplementary Table 1. Initial Hospital-related Associations with SARS-CoV2 -related AKI. These therapies or complications occur within the first 7 days of hospitalization when SARS-CoV2-related AKI is defined. Data presented as number (column percentiles), except where specified. ACE-I = angiotensin-converting enzyme-inhibitors; AKI = acute kidney injury; ARB = angiotensin receptor blockers; IVIG = intravenous immunoglobulin; NSAID = non-steroidal anti-inflammatory drugs; PRISM = Pediatric Risk of Mortality Score; SOFA = Sequential Organ Failure Assessment. aInitial PRISM score missing for 497 pediatric patients. Baseline SOFA score missing for 2741 adult patients; maximum SOFA score missing for 2016 adult patients. [file 12882_2022_2681_MOESM1_ESM.pdf]

Supplementary Table 1. Initial Hospital-related Associations with SARS-CoV2 -related AKI.

	Total	No AKI	AKI (total)
	**6874**	**4155 (60.5)**	**2719 (39.6)**
Co-infections			
Bacterial (blood, sputum, urine)	1092 (16)	474 (11)	618 (23)
Respiratory viral	54 (0.8)	36 (0.9)	18 (0.7)
Initial PRISM score, median (IQR)[a]	7 (4, 11)	5.5 (1, 10)	9 (5, 14)
Baseline SOFA score, median (IQR)[a]	2 (0, 5)	1 (0, 3)	4 (2, 7)
Max SOFA score, median (IQR)[a]	4 (1, 7)	2 (0, 5)	6 (3, 10)
Blood Type			
Group O	779 (11)	417 (10)	362 (13)
Group A	721 (11)	411 (10)	310 (11)
Group B	378 (6)	251 (6)	127 (5)
Group AB	170 (3)	124 (3)	46 (2)
Unknown	4826 (70)	2952 (71)	1874 (69)
Medications/Infusions			
Steroids	2312 (34)	1397 (34)	915 (34)
Remdesivir	1121 (16)	806 (19)	315 (12)
Azithromycin	2399 (35)	1438 (35)	961 (35)
Aspirin	158 (2)	64 (2)	94 (4)
IVIG	142 (2)	72 (2)	70 (3)
Convalescent plasma	268 (4)	144 (4)	124 (5)
Packed red blood cells	174 (3)	55 (1)	119 (4)
ACE-I/ARBs	281 (4)	163 (4)	118 (4)
Diuretics	1369 (20)	593 (14)	776 (29)
NSAIDs	87 (1)	62 (2)	25 (0.9)
Iohexol contrast	1719 (25)	1115 (27)	604 (22)

These therapies or complications occur within the first 7 days of hospitalization when SARS-CoV2-related AKI is defined. Data presented as number (column percentiles), except where specified. ACE-I=angiotensin-converting enzyme-inhibitors; AKI=acute kidney injury; ARB=angiotensin receptor blockers; IVIG=intravenous immunoglobulin; NSAID=non-steroidal anti-inflammatory drugs; PRISM=Pediatric Risk of Mortality Score; SOFA=Sequential Organ Failure Assessment.

[a]Initial PRISM score missing for 497 pediatric patients. Baseline SOFA score missing for 2741 adult patients; maximum SOFA score missing for 2016 adult patients.
