# Supplementary material for: SARS-CoV-2 infection increases risk of acute kidney injury in a bimodal age distribution
Source: BMC Nephrol. 2022 Feb 11;23:63. doi: 10.1186/s12882-022-02681-2 (PMC8831033; doi:10.1186/s12882-022-02681-2)
Supplement: Supplementary file 2 — Additional file 2: Supplementary Fig. 1. Age Distribution of Hospitalized Patients with SARS-CoV2 who Experienced AKI within First 7 days of Hospitalization-different baseline creatinine estimators. Main figure presents percentage per age bracket who developed acute kidney injury (AKI) among all hospitalized patients. The original AKI definition (blue) assumes a baseline creatinine based on KDIGO guidelines for adults (eGFR 75 ml/min/1.73m2 and back calculates using MDRD equation) and common pediatric definitions assuming an eGFR of 120 ml/min/1.73m2 and back calculating using height-independent equation, except for patients with CKD when minimum serum creatinine during first 7 days of hospitalization is assumed to be their baseline creatinine value. Orange line assumes that the minimum creatinine during the first 7 days of hospitalization is the baseline creatinine for all participants. Gray line uses the KDIGO guidelines but back calculates the baseline creatinine for all participants using the FAS equation. Yellow line uses the original definition but uses the MDRD equation minus the race variable. Abbreviations: AKI = acute kidney injury, CKD = chronic kidney disease, eGFR = estimated glomerular filtration rate, FAS = full age spectrum, KDIGO=Kidney Disease Improving Global Outcomes, MDRD = modification of diet in renal disease. [file 12882_2022_2681_MOESM2_ESM.pdf]

Supplementary Figure 1. Age Distribution of Hospitalized Patients with SARS-CoV2 who Experienced AKI within First 7 days of Hospitalization- different baseline creatinine estimators

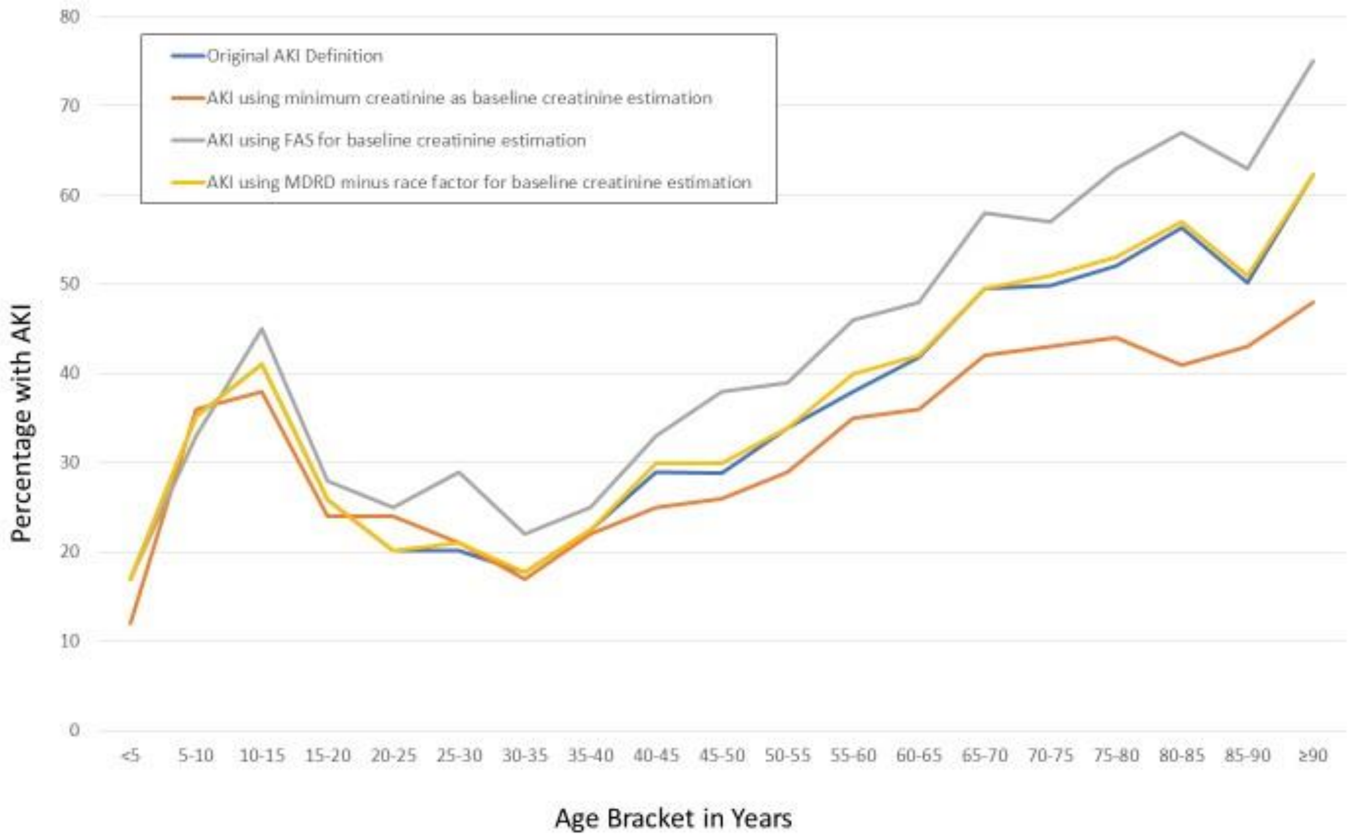

Main figure presents percentage per age bracket who developed acute kidney injury (AKI) among all hospitalized patients. The original AKI definition (blue) assumes a baseline creatinine based on KDIGO guidelines for adults (eGFR 75ml/min/1.73m^2 and back calculates using MDRD equation) and common pediatric definitions assuming an eGFR of 120ml/min/1.73m^2 and back calculating using height-independent equation, except for patients with CKD when minimum serum creatinine during first 7 days of hospitalization is assumed to be their baseline creatinine value. Orange line assumes that the minimum creatinine during the first seven days of hospitalization is the baseline creatinine for all participants. Gray line uses the KDIGO guidelines but back calculates the baseline creatinine for all participants using the FAS equation. Yellow line uses the original definition but uses the MDRD equation minus the race variable.

Abbreviations: AKI=acute kidney injury, CKD=chronic kidney disease, eGFR=estimated glomerular filtration rate, FAS=full age spectrum, KDIGO=Kidney Disease Improving Global Outcomes, MDRD=modification of diet in renal disease
