# Supplementary material for: SARS-CoV-2 infection increases risk of acute kidney injury in a bimodal age distribution
Source: BMC Nephrol. 2022 Feb 11;23:63. doi: 10.1186/s12882-022-02681-2 (PMC8831033; doi:10.1186/s12882-022-02681-2)
Supplement: Supplementary file 5 — Additional file 5: Supplementary Fig. 4. Age Distribution of Hospitalized Patients with SARS-CoV2 who Experienced AKI within First 7 days of Hospitalization-baseline creatinine estimator as minimum serum creatinine. Main figure presents percentage per age bracket who developed acute kidney injury (AKI) among all hospitalized patients and further stratified by severity of illness status. Severe illness is defined as a composite indicator of invasive ventilation, use of vasopressor(s)/inotrope(s), and/or use of extracorporeal membrane oxygenation. Moderate illness is defined as admitted to an intensive care unit but without use of above organ support measures. Mild illness is defined as patient required hospitalization but not in an intensive care unit and without use of above organ support measures. Insert presents the adjusted odds ratio (OR) with 95% confidence intervals (CI) of developing AKI within the first week by age bracket compared to young adults (30–35-year-olds) as the referent category. Adjusted for sex, race/ethnicity, pre-existing hypertension, diabetes mellitus, cancer, chronic kidney disease, and severity of illness. AKI defined per KDIGO guidelines, but baseline creatinine estimator uses minimum serum creatinine value within first 7 days of hospitalization for all participants. [file 12882_2022_2681_MOESM5_ESM.pdf]

Supplementary Figure 4. Age Distribution of Hospitalized Patients with SARS-CoV2 who Experienced AKI within First 7 days of Hospitalization-baseline creatinine estimator as minimum serum creatinine

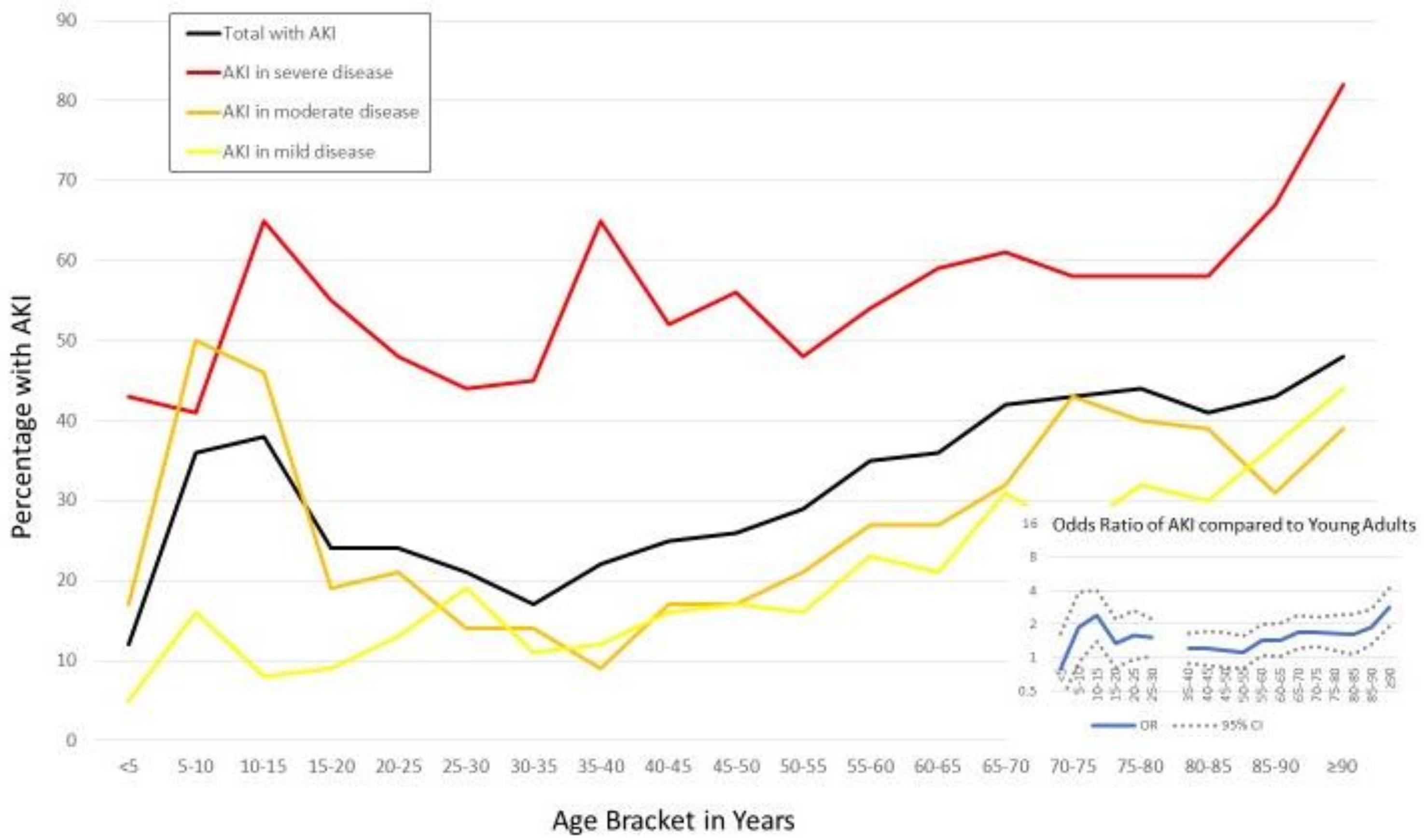

Main figure presents percentage per age bracket who developed acute kidney injury (AKI) among all hospitalized patients and further stratified by severity of illness status. Severe illness is defined as a composite indicator of invasive ventilation, use of vasopressor(s)/inotrope(s), and/or use of extracorporeal membrane oxygenation. Moderate illness is defined as admitted to an intensive care unit but without use of above organ support measures. Mild illness is defined as patient required hospitalization but not in an intensive care unit and without use of above organ support measures. Insert presents the adjusted odds ratio (OR) with 95% confidence intervals (CI) of developing AKI within the first week by age bracket compared to young adults (30-35-year-olds) as the referent category. Adjusted for sex, race/ethnicity, pre-existing hypertension, diabetes mellitus, cancer, chronic kidney disease, and severity of illness. AKI defined per KDIGO guidelines, but baseline creatinine estimator uses minimum serum creatinine value within first seven days of hospitalization for all participants.
