# Supplementary material for: SARS-CoV-2 infection increases risk of acute kidney injury in a bimodal age distribution
Source: BMC Nephrol. 2022 Feb 11;23:63. doi: 10.1186/s12882-022-02681-2 (PMC8831033; doi:10.1186/s12882-022-02681-2)
Supplement: Supplementary file 6 — Additional file 6: Supplementary Fig. 5. Age Distribution of Hospitalized Patients with SARS-CoV2 who Experienced AKI within First 7 days of Hospitalization Stratified by U.S. versus non-U.S. Hospital. Presents percentage of hospitalized patients who developed acute kidney injury (AKI) among all hospitalized patients and further stratified by hospital center based in the United States versus not in the United States. AKI defined per KDIGO guidelines. [file 12882_2022_2681_MOESM6_ESM.pdf]

Supplementary Figure 5. Age Distribution of Hospitalized Patients with SARS-CoV2 who Experienced AKI within First 7 days of Hospitalization Stratified by U.S. versus non-U.S. Hospital.

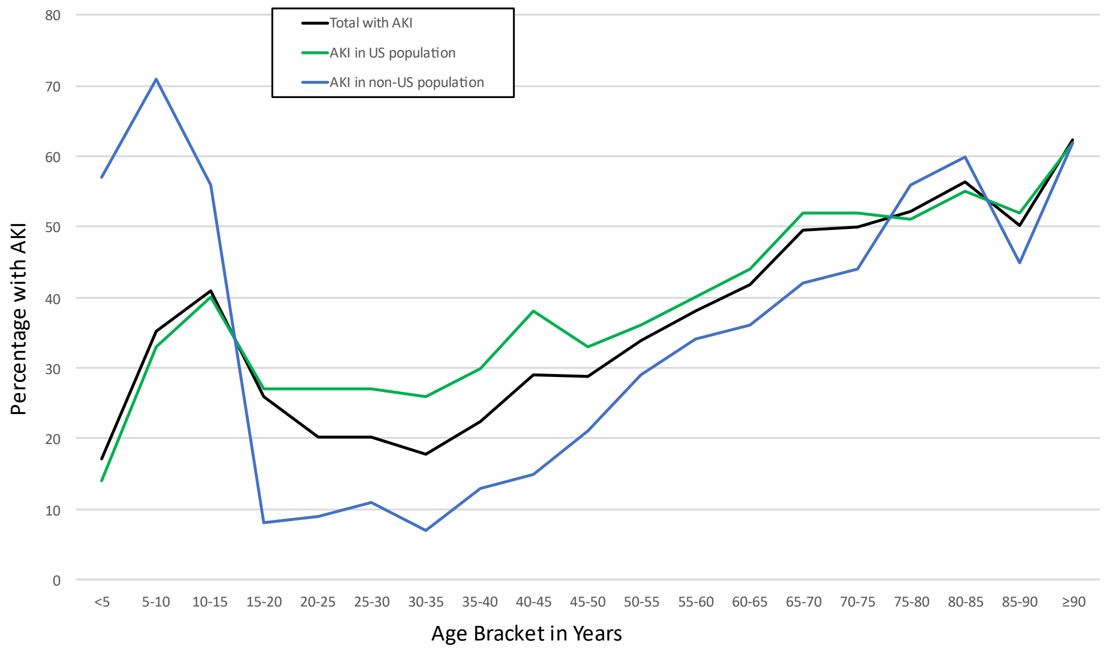

Presents percentage of hospitalized patients who developed acute kidney injury (AKI) among all hospitalized patients and further stratified by hospital center based in the United States versus not in the United States. AKI defined per KDIGO guidelines.
